# Visual Odometry and Place Recognition Fusion for Vehicle Position Tracking in Urban Environments

**DOI:** 10.3390/s18040939

**Published:** 2018-03-22

**Authors:** Safa Ouerghi, Rémi Boutteau, Xavier Savatier, Fethi Tlili

**Affiliations:** 1 Carthage University, SUP’COM, GRESCOM, El Ghazela 2083, Tunisia; fethi.tlili@supcom.tn; 2 Normandie University, UNIROUEN, ESIGELEC, IRSEEM, 76000 Rouen, France; remi.boutteau@esigelec.fr (R.B.); xavier.savatier@esigelec.fr (X.S.)

**Keywords:** real-time navigation, visual-odometry, SeqSLAM, loop-closure, EKF, UKF

## Abstract

In this paper, we address the problem of vehicle localization in urban environments. We rely on visual odometry, calculating the incremental motion, to track the position of the vehicle and on place recognition to correct the accumulated drift of visual odometry, whenever a location is recognized. The algorithm used as a place recognition module is SeqSLAM, addressing challenging environments and achieving quite remarkable results. Specifically, we perform the long-term navigation of a vehicle based on the fusion of visual odometry and SeqSLAM. The template library for this latter is created online using navigation information from the visual odometry module. That is, when a location is recognized, the corresponding information is used as an observation of the filter. The fusion is done using the EKF and the UKF, the well-known nonlinear state estimation methods, to assess the superior alternative. The algorithm is evaluated using the KITTI dataset and the results show the reduction of the navigation errors by loop-closure detection. The overall position error of visual odometery with SeqSLAM is 0.22% of the trajectory, which is much smaller than the navigation errors of visual odometery alone 0.45%. In addition, despite the superiority of the UKF in a variety of estimation problems, our results indicate that the UKF performs as efficiently as the EKF at the expense of an additional computational overhead. This leads to the conclusion that the EKF is a better choice for fusing visual odometry and SeqSlam in a long-term navigation context.

## 1. Introduction

Autonomous vehicles have recently received great attention in the robotics, intelligent transportation, and artificial intelligence communities. Accurate estimation of a vehicle’s location is a key capability to realizing autonomous operation. Currently, the leading technology in this setting is GPS receivers to estimate their absolute, georeferenced pose. However, most commercial GPS systems suffer from limited precision and are sensitive to multipath effects (e.g., in the so-called “urban canyons” formed by tall buildings), which can introduce significant biases that are difficult to detect in addition to sometimes being unavailable (e.g., in tunnels). To provide alternatives to GPS localization, many sensory devices have been applied to carry out the localization task, including visual sensors that are often desirable due to their low-cost, wide availability and passive nature.

Vision-based localization techniques fall into several broad categories including real-time Structure from motion (SfM) or Visual Odometry (VO) and Place Recognition. While VO methods calculate the egomotion by incrementally estimating the rotation and translation undergone by the vehicle using only the input of a single or multiple cameras, Place Recognition methods are based on learning a database of images (the map) and the vehicle consecutively tries to find matchings between this database and the actual visual input (query image(s)). Within the place recognition paradigm, numerous research papers have addressed visual appearance-based place recognition by ground vehicles especially in varying environments [1,2,3,4,5]. A leading method is SeqSLAM aiming at matching image sequences under strong seasonal and illumination changes through the computation of image-by-image dissimilarity scores between all query and database images. Despite its performance under strong changes, SeqSLAM tends to fail in correctly matching images when dealing with changes in viewpoint that can be found between them [6].

Hence, our motivation is to create a vision-driven localization method in urban environments that uses cues from SeqSLAM and VO. Our method is able to localize vehicles in the global reference system if a georeferenced database of images has been learned beforehand. However, in this paper, we address the problem of long-term navigation where loop closures, referring to when the vehicle has returned to a past location after having discovered new terrain for a while, are used to reduce the drift caused by VO. Such detection makes it possible to increase the precision of the actual pose estimate. To this end, we present the integration of the robust sequence-based recognition capabilities of the SeqSLAM system with the accurate 3D metric properties of monocular VO in a probabilistic framework through the use of the Extended Kalman Filter (EKF), the efficient recursive estimator [7] and the unscented Kalman filter (UKF). Keeping power-, space- and weight-constrained applications in mind, we prefer to avoid additional sensors and to utilize only visual cues available in monocular sequences. In fact, despite years of research, monocular-based-localization systems are still an exciting open problem.

The paper proceeds as follows: Section 2 presents some related work. Section 3 summarises the SeqSLAM’s main components and its improved version. Then, our approach details including the position tracking, the uncertainty estimation and the fusion through the filtering techniques are presented in Section 4. Finally, Section 5 presents the results, discusses the outcomes of this paper and presents suggestions for future work.

## 2. Related Work

Several approaches have been developed in the past few years, which frame self-localization as a retrieval task. Towards this goal, multiple representations of the world have been adopted, namely, visual Bag-of-Words (BoW), visual features and 3D point clouds. BoW representation has been first used by the popular algorithm FAB-MAP [8] that relies on extracting scale-invariant image keypoints and descriptors. The descriptor vectors are then quantized using a dictionary trained on prior data. Fab-Map achieves robust image recall performance for outdoor image sequences up to 1000 km [9]. Other approaches dealing with visual features either use a trained georeferenced map of simple visual 3D features [10], or visual features from 3D building geometry [11]. In the case of 3D point clouds, the localization is performed by retrieving point clouds which are similar to the current scene [12].

However, the success of visual features-based approaches is very dependent on the quality of the visual vocabulary, and in turn on the prior data and the reliability of extracting the same visual keypoints and descriptors in images with similar viewpoints. The latter is particularly problematic when there are large lighting variations and scene appearance changes due either to seasonal changes or to day and night cycles. Significant performance improvements over Fab-MAP under extreme lighting and atmospheric variations have been achieved within the SeqSLAM system, which relies on the use of a whole image regardless of its content by searching in a pre-learnt database of images for the most similar sequence to the query sequence [1,2]. SeqSLAM proved its success with very low resolution imagery [2,13], on large-scale environments of 3000 km over four seasons [6], with UV imagery [4] and with ConvNet features [5]. However, one of SeqSLAM’s most significant drawbacks is its lack of viewpoint invariance inherited from the use of global image matching.

On the other hand, when the initial position is known, self-localization is achieved by visual odometry that yields relative motion estimates, integrated to obtain an estimate of the vehicle’s current position. High accuracy has been achieved using stereo-based SfM systems [14]. Good performance of monocular systems has also been demonstrated on the KITTI visual odometry benchmark [15], for example in [16,17], but the incremental nature of these methods inevitably leads to drift. Simultaneous Localization and Mapping (SLAM) methods attempt to reduce this drift by using landmarks and jointly optimizing over all or a selection of poses and landmarks [18,19]. Drift can be further reduced by revisiting places several times and detecting loop closures in the traveled trajectory [20].

However, SLAM methods suffer from issues in terms of speed and map size which limit their application at large scales. Even though efficient optimization strategies using either incremental sparse matrix factorization [21] or relative representations [22] have been used, several recent works have instead relied on publicly available maps [23,24], road networks [25] or satellite images [26] to address the localization of ground vehicles.

Other approaches are based on the fusion of two or multiple algorithms. In fact, data from different algorithms might be combined to derive a more accurate estimate of the vehicle’s pose, as their uncertainties might be complementary. Illustrated in [27] is the fusion of visual odometry, used to track the position of a camera-equipped Micro Aerial Vehicle (MAV) flying in urban streets with an air-ground image matching algorithm using a cadastral 3D city model by means of a Kalman filter. Additionally, our approach is based on the fusion of VO and the appearance-based place recognition algorithm SeqSLAM. We adapted SeqSLAM for use in the context of long-term navigation for loop closure detection in order to reduce the drift caused by VO. The database of images is, therefore, created online. However, in the case of learning a geo-referenced database beforehand, the method remains valid and more accurate estimates could be obtained in the global reference frame without assuming a loopy trajectory.

## 3. Appearance-Based Global Positioning System

In this section, we briefly present the main components of the state-of-the-art appearance-based global positioning algorithm SeqSLAM and then show some components of the Sequence Matching Across Route Traversals (SMART) system, built upon SeqSLAM to overcome some of its limitations. The SeqSLAM algorithm, using a simple whole-image comparison with Sum of Absolute Differences (SAD), demonstrated impressive place recognition performance across significant condition variance such as seasonal changes and day to night transitions where feature-based methods failed entirely and in the case of low quality imagery (low resolution, low depth, and image blur). In order to increase the discriminative nature of the observation and to avoid the problem of false-positives, location is represented in SeqSLAM as a sequence of images, rather than a single image from one pose. Images are, beforehand, resolution-reduced and patch-normalised to enhance contrast and are, therefore, converted into visual templates. Then, to compare each query image $I_{q}$ from the query sequence $Q=(I_{1},\ldots ,I_{Q})$ where Q=|Q| to each database image $I_{D}$ from the database $D=(I_{1},\ldots ,I_{D})$ where D=|D|, SeqSLAM calculates the difference score *d*

(1) $$d=\frac{1}{R_{x}R_{y}}\left|I_{Q}-I_{D}\right|$$

where $R_{x}$ and $R_{y}$ are the horizontal and vertical image dimensions, respectively. The difference scores are assembled into the so-called difference matrix.

**Figure 1:**
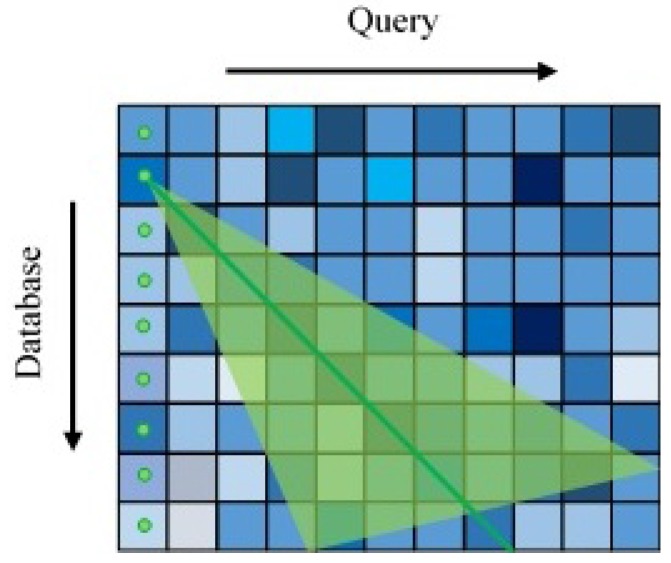
For each matrix entry, a sub-route score for a range of possible slopes (semi-transparent region). When all scores are calculated for one starting point, the minimal score of all of them is selected. Finally, the sub-route with the smallest score and the second smallest score are used to compute the best database matching to the input query sequence.

A next key processing step is to normalize the image difference values (d) within their (spatially) local image neighborhoods. Subsequently, a search for diagonals of low difference values is performed over the defined sequence length (Q) as depicted in Figure 1.

**Figure 2:**
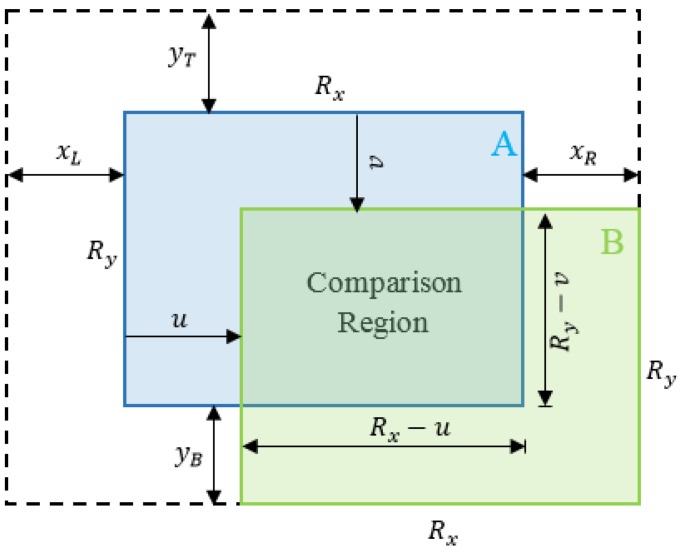
For each comparison, query frame B is slid over template frame A in a range of offsets (within the dashed boundary), with the minimum calculated difference score.

However, one of SeqSLAM’s most significant drawbacks is its lack of viewpoint invariance inherited from the use of global image matching. To compensate for a small viewpoint invariance, variable offset image matching and distance-based template learning have been used in the SMART system built upon SeqSLAM [28]. In fact, to improve performance on traverses with small shifts in lateral pose, a variable offset image matching is used where each query frame (template) is compared to each database frame (template) at a range of offsets with the sum of absolute differences (SAD) performed on the overlapping region and the minimum difference score is used:

(2) $$d=\min_{\begin{array}{c} x_{max\;left}\leq u\leq x_{max\;right}\\ y_{max\;up}\leq v\leq y_{max\;down} \end{array}}d\left(u,v\right)$$

(3) $$d\left(u,v\right)=\frac{1}{|X_{A}\left|\right|Y_{A}|}\sum_{\begin{array}{c} x_{a}\in X_{A}\\ x_{b}\in X_{B} \end{array}}\sum_{\begin{array}{c} y_{a}\in Y_{A}\\ y_{b}\in Y_{B} \end{array}}\left|A_{x_{a},y_{a}}-B_{x_{b},y_{b}}\right|$$

where *u* and *v* are the coordinates in the difference score, $X_{A},\;Y_{A},\;X_{B}$ and $Y_{B}$ are vectors representing the overlapping region of images A and B and $x_{a}$ and $x_{b}$ are the pixel coordinates in A and B respectively (Figure 2).

On the other hand, SMART learns (creates the database of templates) and queries templates at regular distance intervals (fixed distance $f_{dist}$ between templates) rather than fixed time or frame intervals as SeqSLAM does (fixed number of frames between database and query templates). To function effectively, odometry information (sourced either from wheel encoders or vision) is needed [28].

## 4. Proposed Approach

**Figure 3:**
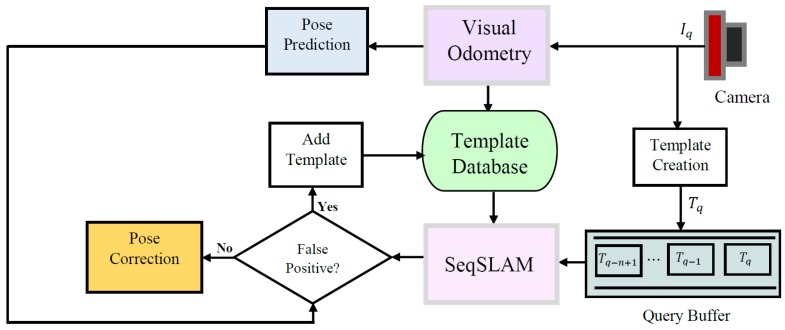
Block diagram of the main components of the system.

We propose a kind of hybrid approach that combines local metrical localization (e.g., visual odometry) with a topological one (e.g., SeqSLAM). The vehicle tracking is performed by VO leading to drift due to the run-time error accumulation. Loop closure recognition via SeqSLAM allows incremental pose drift [29] to be overcome and the state and position of the vehicle to be recovered in cases where the tracking is lost. The use of a two algorithms fusion-based approach is mainly motivated by the fact that VO is prone to drift and place recognition approaches generally suffer from false positive (FP) detections. Although false positives have an impact on the short-term deviation of the system inside the filtering framework, this latter robustly recovers after some time. Combined methods give, therefore, an increased robustness to the system. The template library can be loaded beforehand (when a learning has already been done) or created in real-time (without prior learning). In such a case, the template database is populated with the acquired frames from the monocular stream converted into visual templates along with their poses acquired from the VO module. The query sequence is a FIFO buffer holding the *n* last templates. In fact, when a new frame is captured, features are detected and matched with the last acquired frame and the relative motion is estimated using the VO module, which will subsequently allow the pose of the vehicle to be predicted. The frame is also converted into a visual template and added to the query sequence with the aim of matching with a database template. The SeqSLAM module performs the matching that will be tested to determine whether it is a true positive based on both a matching score provided by SeqSLAM and the prediction made by the VO module. If a place is recognized, a correction of the pose is made. Otherwise, the template is assumed to belong to a new place and is, therefore, added to the database as depicted in Figure 3.

### 4.1. Position Tracking

The goal of this section is to track the state of the vehicle over several images. The vehicle state in time *k* is composed by the position in the 2D plane and the orientation with respect to the first reference frame. Thus, we consider the reduced state vector $x_{k}\in R^{3}$

(4) $$x_{k}=\left(p_{k},\theta_{k}\right),$$

where $p_{k}\in R^{2}$ denotes the position and $\theta_{k}\in R$ denotes the orientation (heading angle).

We adopt a Bayesian approach [30] to track and update the position of the vehicle. We compute the posterior probability density function *(pdf)* of the state in two steps. To compute the prediction update of the Bayesian filter, we use VO. To compute the measurement update, we integrate the topological localization update, whenever it is supplied by SeqSLAM, described in the previous section.

The system model *f* describes the evolution of the state over time. The measurement model *h* relates the current measurement $z_{k}\in R^{3}$ to the state. Both are expressed in a probabilistic form:

(5) $$x_{k|k-1}=f\left(x_{k-1|k-1},u_{k-1}\right),$$

(6) $$z_{k}=h\left(x_{k|k-1}\right),$$

where $u_{k-1}\in R^{3}$ denotes the output of the VO algorithm at time k−1, $x_{k|k-1}$ denotes the prediction of estimate *x* at time *k* and $x_{k-1|k-1}$ denotes the updated estimate of *x* at time k−1. The functions *f* and *h* are in general non-linear functions.

#### 4.1.1. Visual Odometry System

Visual Odometry (VO) is usually referred to as the problem of incrementally estimating the egomotion of a vehicle using a single or multiple cameras [31]. In order to deal with all central camera models including perspective, dioptric, omnidirectional and catadioptric imaging devices, image measurements are represented as 3D bearing vectors: a unit vector originating at the camera center and pointing toward the landmark. Each bearing vector has only two degrees of freedom, which are the azimuth and elevation inside the camera reference frame as formulated in the OpenGV library [32]. Because a bearing vector has only two degrees of freedom, we refer to it as 2D information and it is normally expressed in a camera reference frame. The fundamental matrix solver within the OpenGV library computes the relative pose of a viewpoint with respect to another viewpoint given a number of eight correspondences between bearing vectors expressed in the respective camera frames within a Random Sample Consensus (RANSAC) framework to deal with false matchings [33].

#### 4.1.2. State Prediction and Uncertainty Estimation

At time *k*, two consecutive images $I_{k}$ and $I_{k-1}$ are given as input to the VO algorithm. This latter returns an incremental motion estimate with respect to the local camera reference frame. We define this estimate as $\delta_{k,k-1}^{*}\in R^{3}$

(7) $$\delta_{k,k-1}^{*}=\left(\Delta s_{k}^{*},\Delta \theta_{k}\right),$$

where $\Delta s_{k}^{*}\in R^{2}$ denotes the translational component of the motion and $\Delta \theta_{k}$ the heading angle increment.

As we are using monocular visual odometry using only the input of a single camera, we deal with a scale ambiguity where the norm of the translational component cannot be recovered and we only obtain the direction of the translation. $\Delta s_{k}^{*}$ is valid up to a scale factor and, thus, the metric translation of the vehicle in the ground-plane $\Delta s_{k}\in R^{2}$ at time *k* with respect to the local camera frame is equal to

(8) $$\Delta s_{k}=\lambda \Delta s_{k}^{*},$$

where λ∈R is the scale factor. Several approaches have shown their efficacy in solving the scale factor ambiguity within the monocular scheme including the use of prior knowledge of the camera height relative to ground-plane [34].

Given that we predicted the state of the vehicle $x_{k}$ using $x_{k-1}$ and the incremental motion estimate $\delta_{k,k-1}\in R^{3}$, the uncertainty of the pose has to be estimated as well, represented by a 3×3 covariance matrix.

In order to estimate the covariance matrix $\Sigma_{\delta_{k,k-1}}\in R^{3\times 3}$, we use the Monte Carlo technique [35]. In general, when dealing with a nonlinear function f(.) that relates a random variable *Y* to an N-dimensional random variable *X* such as Y=f(X), where *X* has a mean $\bar{X}$ and a covariance $\Sigma_{X}$, the transformed mean and covariance of *Y* can be obtained via a Monte Carlo simulation that relies on a repeated random sampling. In fact, a large number of samples $\left\{X_{1},\;X_{2}\;\ldots \;X_{n}\right\}$ are randomly drawn from *X* and the function f(.) is evaluated for each sample. For the transferred samples from function f(.), $\left\{Y_{1},\;Y_{2}\;\ldots ,\;Y_{n}\right\}$, the mean $\bar{Y}$ and covariance $\Sigma_{Y}$ are estimated according to:

(9) $$\bar{Y}=\frac{1}{N}\sum_{i=1}^{N}Y_{i}$$

(10) $$\Sigma_{y}=\frac{1}{N-1}\sum_{i=1}^{N}\left(Y_{i}-\bar{Y}\right){\left(Y_{i}-\bar{Y}\right)}^{T}.$$

In the case of VO, the algorithm uses at every step a set of 2D corresponding bearing vectors between images *k* and k−1 and provides an incremental estimate $\delta_{k,k-1}$. In fact, features which are detected and matched between every consecutive pair of frames are, subsequently, converted into bearing vectors. We randomly sample eight correspondences from corresponding bearing vectors and feed them to the RANSAC procedure to compute an estimate $\{\delta_{i}\}.$ All the estimates for which the number of inliers exceeds a prefixed threshold *t* are saved in a set $S=\{\delta_{i}\}$. Estimating the covariance matrix using the Monte Carlo technique requires a large number of samples to be randomly drawn from the initial N-dimensional random variable *x*. We usually obtain a high number of valid Monte Carlo estimates (e.g., more than 500 out of the 1000 iterations result in a valid estimation) and the covariance $\Sigma_{\delta_{k,k-1}}$ is, finally, estimated using (9) and (10).

The error of the VO is propagated throughout consecutive camera positions as follows. At time *k*, the state $x_{k|k-1}$ depends on $x_{k-1|k-1}$ and $\delta_{k,k-1}$

(11) $$x_{k|k-1}=f\left(x_{k-1|k-1},\delta_{k,k-1}\right).$$

We compute the associated covariance $\Sigma_{x_{k|k-1}}\in R^{3\times 3}$ by the error propagation law:

(12) $$\Sigma_{x_{k|k-1}}=\nabla f_{x_{k-1|k-1}}\Sigma_{x_{k-1|k-1}}\nabla f_{x_{k-1|k-1}}^{T}+\nabla f_{\delta_{k,k-1}}\Sigma_{\delta_{k,k-1}}\nabla f_{\delta_{k,k-1}}^{T}$$

assuming that $x_{k-1|k-1}$ and $\delta_{k,k-1}$ are uncorrelated.

We compute the Jacobian matrices numerically, the rows of the jacobian matrices $\nabla {(}^{i}f_{x_{k-1|k-1}}),$ $\;\nabla {(}^{i}f_{\delta_{k,k-1}})\in R^{1\times 3}\;\left(i=1,\;2,\;3\right)$ are computed as:

(13) $$\nabla {(}^{i}f_{x_{k-1|k-1}})=\left[\frac{\partial {(}^{i}f)}{\partial {(}^{1}x_{k-1|k-1})}\;\frac{\partial {(}^{i}f)}{\partial {(}^{2}x_{k-1|k-1})}\;\frac{\partial {(}^{i}f)}{\partial {(}^{3}x_{k-1|k-1})}\right],$$

(14) $$\nabla {(}^{i}f_{\delta_{k|k-1}})=\left[\frac{\partial {(}^{i}f)}{\partial {(}^{1}\delta_{k,k-1})}\;\frac{\partial {(}^{i}f)}{\partial {(}^{2}\delta_{k,k-1})}\;\frac{\partial {(}^{i}f)}{\partial {(}^{3}\delta_{k,k-1})}\right],$$

where ${}^{i}x_{k-1|k-1}$ and ${}^{i}\delta_{k,k-1}$ denote the *i*-*th* component of $x_{k-1|k-1}$ and $\delta_{k,k-1}$ respectively.

### 4.2. Loop Closure-Based Measurement Update

**Figure 4:**
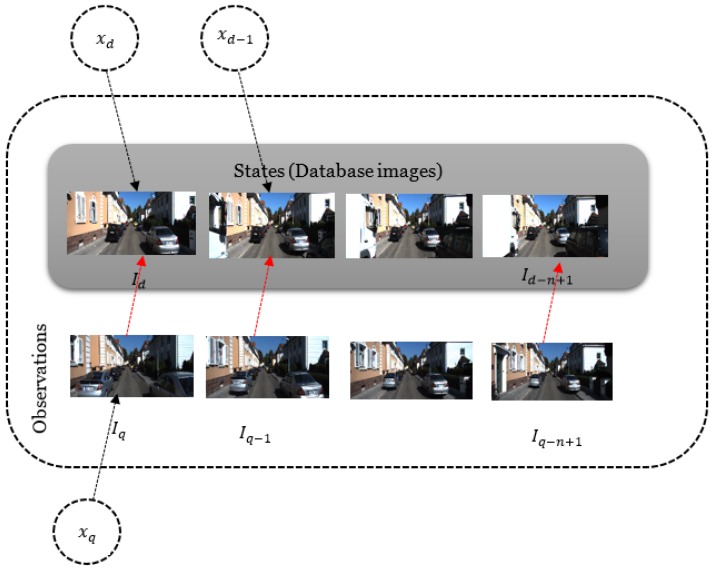
Unobservable state variables for a measurement update obtained from observable query and database templates by a linear model.

In the SeqSLAM algorithm, the input is the query sequence that refers to the last acquired images converted to visual templates. The algorithm selects the path in the difference matrix that connects the query templates to the database ones. The observations are the sequence of *n* query images $I_{q}$, and the state space *S* is the set of database images $I_{d}$. For each observation there is a state corresponding to one of the database images in the state space as depicted in Figure 4 where primitive high-resolution images are used instead of low-resolution templates for the purpose of clarity. Thus, the observables here are the query and database templates, which are continuously calculated from the monocular sequence and the tracked state $x_{k}=\left(x,y,\theta \right)$ is hidden. However, to each database template is connected a pose where this image has been taken and to the query template to be matched is connected the predicted state. Hence, the unobservable state variables are directly obtained from the observable templates and the transformation from the state space to the observation space is a simple linear model.

(15) y=Hx.

where $H=I_{3}$. In fact, when a place is recognized, the corresponding information of the node (matched in the database) can be used as an observation of the filter.

### 4.3. False Positives Filtering

The Gaussian assumption underlying the Kalman Filter and its variants implies that when an observation that significantly differs from the state estimate, as determined from the state covariances, is incorporated into the state estimate, the resulting Gaussian deviates significantly from its prior shape. This means that the current estimate of the true state is no longer useful which has a negative impact on the system robustness. Although the filter recovers after some time, several applications depend on the short-term result in which false positives (FP) have a disastrous impact. In fact, even with the use of a sequence matching instead of a single image by the SeqSLAM algorithm, this latter still suffers from false-positives. Thus, to filter them out, we use the state and covariance estimates and the Gaussian basis of filtering techniques to estimate the likelihood of a given observation. The conditional *pdf* $P(z_{k}|x_{k},\Sigma_{x_{k}})$ is evaluated for the given observation and state estimates to reject observations with too low likelihood as false-positives. The conditional *(pdf)* is simply a multi-dimensional Gaussian with the mean the estimated state and its covariance. Both the mean and the covariance have to be transformed into the observation space. Hence, the likelihood has the following form

(16) $$P\left(z_{k}|x_{k},\Sigma_{x_{k}}\right)=\frac{1}{\sqrt{{\left(2\pi \right)}^{n}C_{k}}}e^{-1/2{\left(z-Hx_{k}\right)}^{T}C_{k}^{-1}\left(z-Hx_{k}\right)}$$

where $C_{k}=H\Sigma_{x_{k}}H^{T}+R$ is the covariance matrix in the observation space, *n* the state dimension and *R* is the measurement covariance matrix. If the evaluated likelihood is above a preset, empirically determined threshold, the measurement update step is performed. Otherwise, only the prediction is done and the covariance of the state estimate will grow until a successful observation is reported. As well as the case of rejected observations, the dynamics update is still performed in the case of false negatives and they, therefore, do not affect the system performance.

SeqSLAM uses a threshold on similarity between sequences to determine whether a match refers to a true positive determined by precision-recall tests. The lower the threshold is, the more similar are the matched images. However, a too low threshold leads to false negatives (e.g., non-detections). The better compromise has been achieved when using both the similarity score and the likelihood to assess true positives without too many non detections as presented in Algorithm 1.
**Algorithm 1:** False positives filtering.
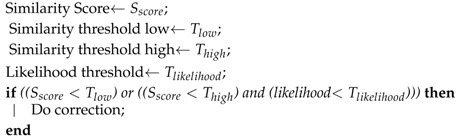


### 4.4. Filtering-Based Fusion of Visual Odometry and SeqSLAM

Our aim is to reduce the uncertainty associated to the state estimate by fusing the prediction estimate with the measurement, whenever a valid measurement issued from a recognized loop closure by SeqSLAM is available. The outputs of this fusion step are the updated estimate $x_{k|k}$ and its covariance $\Sigma_{x_{k|k}}\in R^{3}$. We compute them according to the Kalman filter (KF) update mode as we are dealing with a linear measurement model. The update step is first performed by predicting the measurement and its uncertainty given by

(17) $$z_{k|k-1}=Hx_{k|k-1},$$

(18) $$S_{k}=H\Sigma_{k|k-1}H^{T}+R.$$

Then, the measurement update adjusts the prediction results according to

(19) $$x_{k|k}=x_{k|k-1}+K_{k}\left(z_{k}-z_{k|k-1}\right)$$

(20) $$=\left(I-K_{k}H\right)x_{k|k-1}+K_{k}z_{k}.$$

(21) $$\Sigma_{k|k}=\left(I-K_{k}H\right)\Sigma_{k|k-1}{\left(I-K_{k}H\right)}^{T}+K_{k}RK_{k}^{T}$$

with $K_{k}$ a gain matrix that is optimal in the minimum variance sense and is given by

(22) $$K_{k}=\Sigma_{k|k-1}H^{T}S_{k}^{-1}=M_{k}S_{k}^{-1},$$

where $M_{k}$ is the cross-covariance between the state and output predictions.

### 4.5. Unscented Kalman Filter

The Unscented Kalman Filter (UKF) is an alternative to the Extended Kalman Filter (EKF) demonstrating a superior performance and ease of implementation for nonlinear estimation. The UKF uses an alternative form for dealing with nonlinearity based on the sigma points. The time update includes the weights and sigma points calculations, whereas the measurement update uses the sigma points to generate the covariance matrices and the Kalman gain respectively. The augmented state matrix Y is constructed from the prior belief of the state $\left(x_{k-1|k-1},\Sigma_{k-1|k-1}\right)$:

(23) $$Y=\left[\begin{array}{cc} \Sigma_{k-1|k-1} & 0\\ 0 & Q_{k} \end{array}\right]$$

where $Q_{k}$ is the process noise covariance matrix estimated via a Monte Carlo simulation as explained in the previous section. The prior belief $\left(x_{k-1|k-1},\Sigma_{k-1|k-1}\right)$ is converted into a sigma point representation via:

(24) $$AA^{T}=Y,\;(\mathrm{cholesky}\;\mathrm{decomposition})$$

(25) $$\chi_{0}=x_{k-1|k-1}$$

(26) $$\chi_{i,k|k-1}=x_{k-1|k-1}+\sqrt{(n+\kappa }){\mathrm{col}}_{i}A,\;i=1,\;\ldots ,\;n$$

(27) $$\chi_{i,k|k-1}=x_{k-1|k-1}-\sqrt{(n+\kappa }){\mathrm{col}}_{i}A,\;i=n,\;\ldots ,\;2n$$

where κ is a scaling parameter that affects fourth and higher order moments of the *pdf*. The associated weights $\beta_{i}$ are the following:

(28) $$\beta_{i}=\left\{\begin{array}{cc} \frac{\kappa }{n+\kappa } & i=0\\ \frac{1}{2}\frac{\kappa }{n+\kappa } & \mathrm{otherwise}. \end{array}\right.$$

The subsequent step consists in passing the sigma point through the state evolution model

(29) $$\gamma_{i,k|k-1}=f\left(\chi_{i,k|k-1}\right)\;i=0,\;\ldots ,\;2n.$$

The mean and covariance estimates for γ are calculated as

(30) $$x_{k|k-1}=\sum_{i=0}^{2n}\beta_{i}\gamma_{i,k|k-1},$$

(31) $$\Sigma_{k|k-1}=\sum_{i=0}^{2n}\beta_{i}\left(\gamma_{i,k|k-1}-x_{k|k-1}\right){\left(\gamma_{i,k|k-1}-x_{k|k}\right)}^{T}.$$

The estimate of the posterior $\left(x_{k|k},\Sigma_{k|k}\right)$ is performed through passing each sigma point according to the observation model

(32) $$\upsilon_{i,k|k}=H.\chi_{i,k|k-1},\;i=0,\;\ldots ,\;2n.$$

The predicted measurement and innovation covariance are then computed

(33) $$y_{k|k-1}=\sum_{i=0}^{2n}\beta_{i}\upsilon_{i,k|k},$$

(34) $$V_{k|k}=\sum_{i=0}^{2n}\beta_{i}\left(\upsilon_{i,k|k}-x_{k|k-1}\right){\left(\upsilon_{i,k|k-1}-x_{k|k}\right)}^{T}+R_{k},$$

where $R_{k}$ is the measurement noise covariance matrix. The state measurement covariance and Kalman gain are, next, built according to:

(35) $$U_{k|k}=\sum_{i=0}^{2n}\beta_{i}\left(\chi_{i,k|k-1}-x_{k|k-1}\right){\left(\gamma_{i,k|k}-y_{k|k-1}\right)}^{T},$$

(36) $$K_{k|k}=U_{k|k}V_{k|k}^{-1}.$$

Finally, the posterior belief, $x_{k|k}$, $\Sigma_{k|k}$ is computed according to:

(37) $$x_{k}=x_{k|k-1}+K_{k}\left(y_{k}-y_{k|k-1}\right)$$

(38) $$\Sigma_{k}=\Sigma_{k|k-1}-K_{k}U_{k}^{T}.$$

## 5. Experiments and Results

### 5.1. The Experimental Dataset and Parameters

#### 5.1.1. The Experimental Dataset

The Kitti odometry dataset [15], recorded from cars driven in urban and rural areas and on highways, consists of 22 sequences where the first 11 are provided with ground truth.

**Figure 5:**
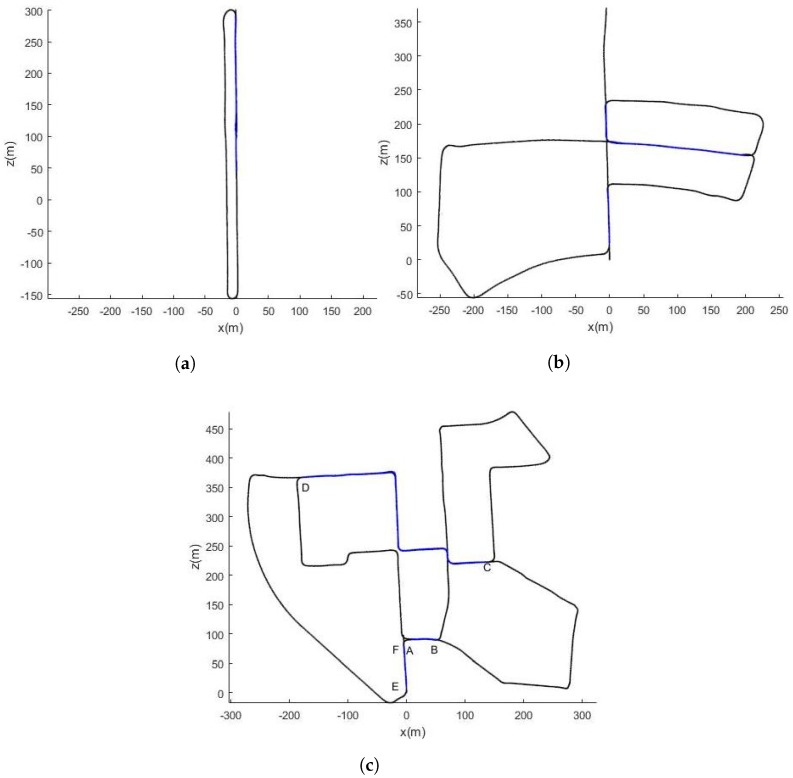
Ground truth trajectories of sequences 00, 05 and 06 of the KITTI dataset comprising loop-closures. (**a**) Sequence 06; (**b**) Sequence 05; (**c**) Sequence 00.

In order to evaluate our method, we conducted experiments on three data sequences from the odometry category with a loopy trajectory. Together, these sequences, presented in Figure 5, include 1249 images of loop closures. The total driving distance for loop closures in these sequences is 1169.6 m and are highlighted in blue in Figure 5. The locations of loop closures for the Sequence 00 in terms of the amount of travelled meters from the first reference pose are presented in Table 1.

We recall that the Kitti dataset provides challenging benchmarks to the computer vision community that are available online www.cvlibs.net/datasets/kitti.

#### 5.1.2. Parameters

The parameters used for SeqSLAM are those reported in the literature and presented in Table 2.

In order to calibrate SeqSLAM and evaluate the uncertainty of the position determination, we used almost 50% of the 1249 images providing two traversals of the same route. The first traversal was used as a database sequence and the second as a query sequence. We assimilated the measurement noise $\nu_{k}=N\left(0,R\right)$. The matching results were compared to the ground truth. In the case of true positives, the deviations have been chosen to be respectively $\sigma_{x}=1$, $\sigma_{y}=1$ and $\sigma_{\theta }=0.1$ so that the returned result is always within 3 standard deviations from the ground truth.

### 5.2. Results

#### 5.2.1. Accuracy Evaluation

**Figure 6:**
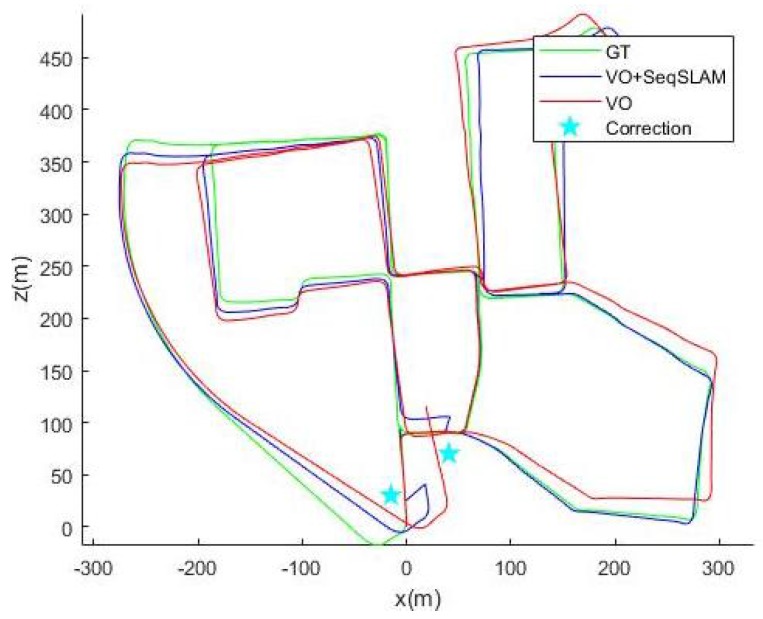
Ground truth, Visual Odometry (VO) and (VO+seqSLAM) for Sequence 00.

**Figure 7:**
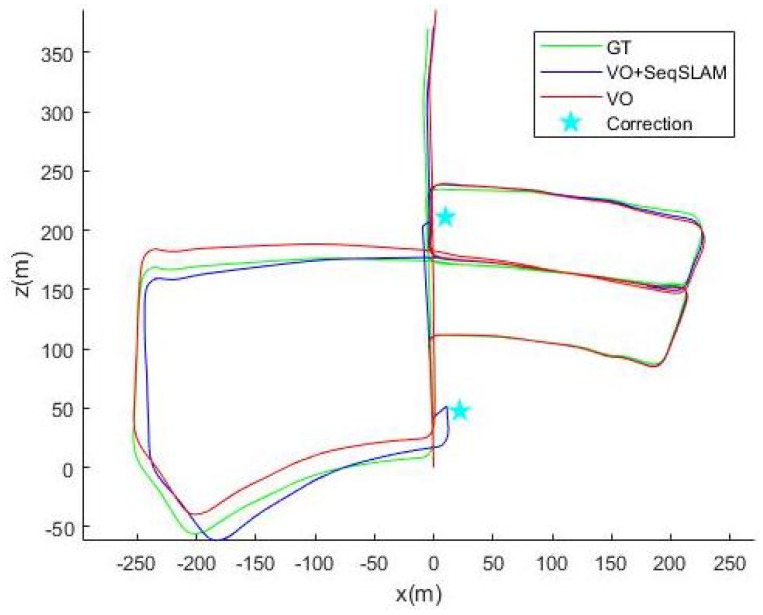
Ground truth, VO and (VO+seqSLAM) for Sequence 05.

**Figure 8:**
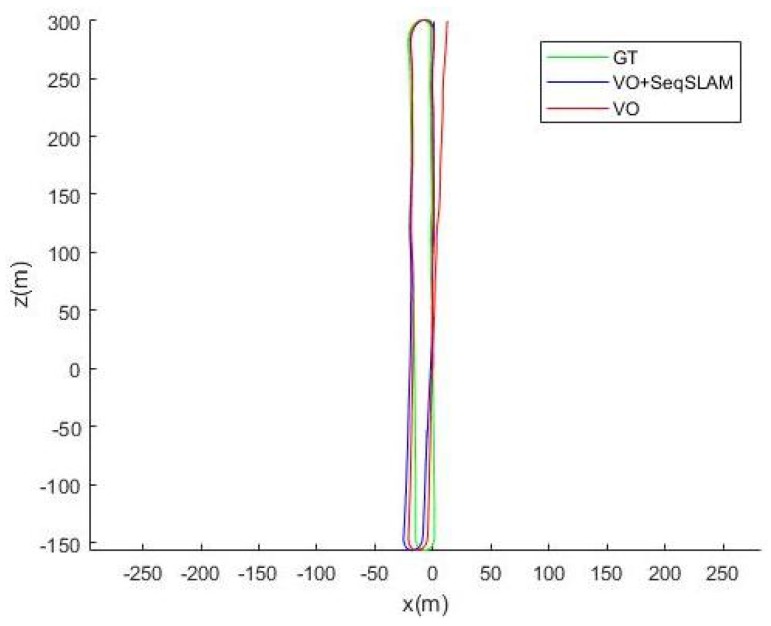
Ground truth, VO and (VO+seqSLAM) for Sequence 06.

We display the ground truth trajectory measured by a precise GPS in green in Figure 6, Figure 7 and Figure 8 for Sequences 00, 05 and 06 respectively from the Kitti odometry benchmark. We show the path estimated by VO in red and the corrected one (VO+SeqSLAM) in blue. Even though the position is generally accurately detected, the non-detections (FN) are sometimes visible (the correction is not made in the beginning of a loop-closure). In fact, when an update from SeqSLAM is integrated into the Bayesian tracking to correct the trajectory after an important drift, the correction is suddenly made, resulting in a non-smooth trajectory (marked by the blue stars). The drift, though, is greatly reduced for a precise localization afterwards.

**Figure 9:**
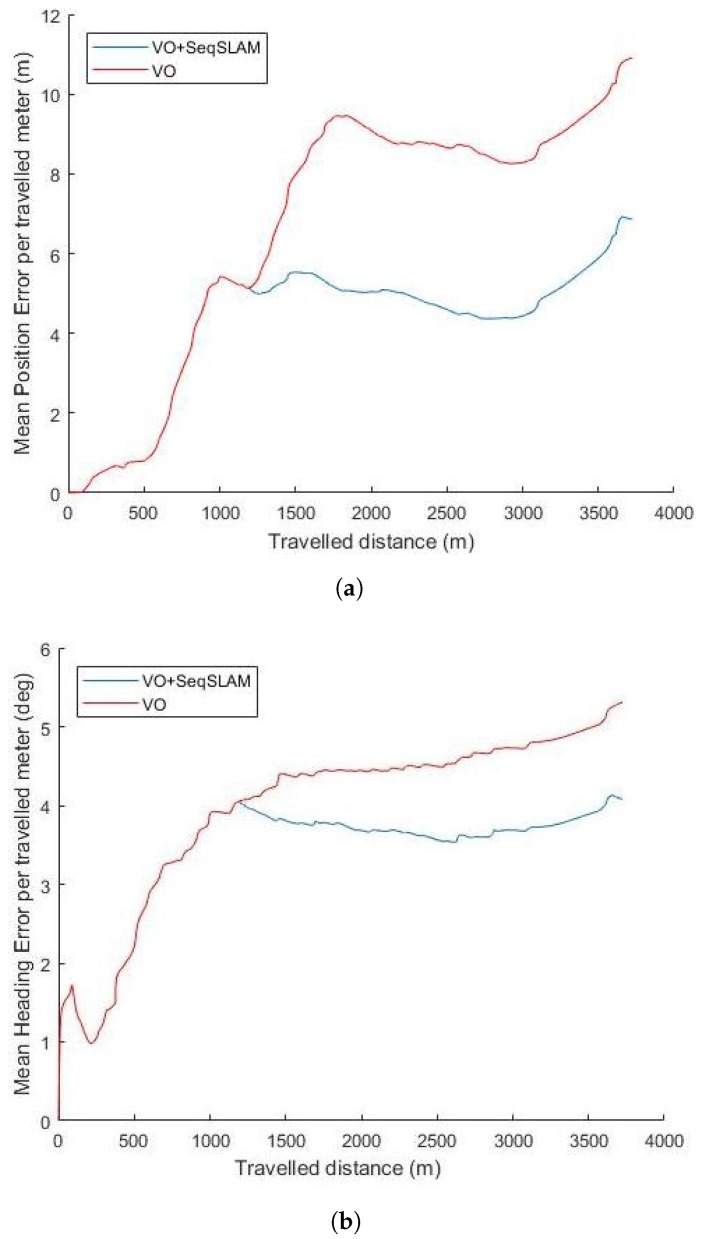
Mean error per travelled meter with respect to the travelled distance for Seq00. (**a**) mean position error per travelled meter; (**b**) mean heading error per travelled meter.

We show the mean error per travelled meter of position and heading in Figure 9 for the Sequence 00. For a travelled trajectory of 1100 m, no loop closures are detected and the mean error in position is 5 (m) and 4 (deg) in heading. For 3000 (m) of travelled distance where two loop closures are detected (Segment AB and Seqment CD in Figure 5c), the mean error is 5 (m) in position and 3.6 (deg) in heading when the SeqSLAM-based correction is integrated into the Bayesian tracking against 9 (m) in position and 4.5 (deg) for VO only. When the vehicle performs a big rotation movement (in segment DE of Figure 5c), the mean error increases dramatically to reach 11 (m) for VO and 6.7 (m) for the SeqSLAM-based corrected VO for a travelled distance of almost 4000 (m). The problem of important drift caused by big rotations is inherent to the monocular VO.

We show the comparative results between the corrected and non-corrected journeys in Table 3 in terms of average error in meters and the percentage of the error with respect to the whole trajectory. The performance of SeqSLAM-based loop closure detection and correction is almost two times better than only VO. We also present in Table 4 the error of the ending pose for all the test sequences.

#### 5.2.2. Fusion Method Evaluation

The UKF is a superior alternative to the EKF for a variety of estimation and control problems. However, its effectiveness depends on the nonlinearity of the problem. In our case, we are dealing with a nonlinear prediction model and a linear measurement model. As listed in Table 5, our experimental results and analysis indicate that UKF performs as efficiently as the EKF. However, the additional computational overhead of the UKF and the linear nature of the update step with SeqSLAM’s observations lead to the conclusion that the EKF is a better choice for fusing VO and SeqsLAM.

### 5.3. Timing and Storage

In this section, we briefly describe the storage and computational requirements of the system. In fact, all the templates are down-sampled from 1241×376 to 64×16 (0.22%) pixels. Consequently, the storage and computational requirements are greatly reduced. To deal with the computational complexity of building a difference matrix between the query and all the database images, a CUDA-based solution was designed in [36], allowing less than 30 ms to be achieved for the query and database sequences used in our experiments. This timing is based on the use of a mid-range GPU, the CUDA NVIDIA GeForce GTX 850M running at 876 MHz with 4096 MB of GDDR device memory. The acceleration is mainly based on the allocation of the three major steps of SeqSLAM, namely: the difference matrix computation, the difference matrix contrast-enhancement and the route searching to three GPU kernels that exploit the parallel CUDA threads and thread-blocks and the fast shared memory. The database, in this design, is stored in the GPU’s global memory. Regarding the timing using a commercially available laptop with an 8 core-2.40 GHz clock, it is around 100 ms.

### 5.4. Discussion

The template library is created online using navigation information from the VO. That is, when there is not a match in the database, a node is added to this latter with the template and the corresponding information of pose obtained from the VO module. The sequence searching strategy used in this work is based on a regular time interval but could be optimized for a regular distance interval between templates to make the localized sequence searching algorithm more efficient as reported in [28]. In fact, we have not relied on the regular distance strategy as we have used the Kitti dataset for evaluation. A regular distance consists in taking templates at a regular distance of 1 m for example. Moreover, this work is a proof of concept that an enhancement could be obtained by only detecting loop closures without jointly optimizing over landmarks and poses as a SLAM system does. However, the greatest interest would be generated when a learning of a geotagged database is already performed which would allow an absolute localization in the global reference frame. The SeqSLAM algorithm could also be adapted to work with different camera systems to make it possible to share the database of templates between different users. Furthermore, even though the drift has been greatly reduced, other methods could be fused with SeqSLAM and VO to make the output suitable for autonomous cars.

## 6. Conclusions

This paper presented a solution to localize a vehicle in urban environments by means of the integration of VO and SeqSLAM. SeqSLAM is a well-known successful approach for place recognition in varying conditions such as seasonal changes and day and night cycles when not dealing with a viewpoint change. The localization was performed using a single on-board camera and the template library was created online using navigation information from the VO. The integration was performed with a Bayesian filtering through the use of an EKF. That is, when a place is recognized, the corresponding information is used as an observation of the filter, otherwise the prediction is performed by the VO module. The performance of the system can be increased by using a pre-learnt geo-tagged database of images to develop a reliable alternative to satellite-based global positioning. In such a case, the importance of SeqSLAM is even more important due to its ability to deal with severe lighting differences between the learnt database and the actual visual input. The results showed the superiority of the visual odometry integrated with SeqSLAM algorithm in real-time localization as the navigation errors are greatly reduced by loop-closure detection.

## Figures and Tables

**Table 1:** Loop closure locations in meters for Sequence 00.

Sequence	Distance up to	Traveled Distance (m)
	A	1160.1
	B	1204.8
00	C	2566.1
	D	3023.3
	E	3636.3
	F	3707

**Table 2:** SeqSLAM’s parameters.

Parameter	Value	Description
$R_{x}$, $R_{y}$	64,16	Template size
*Q*	10	Query sequence length
*P*	8×8 pixels	Patch normalization size
$X_{L},X_{R},Y_{T},Y_{R}$	1,1,1,1	Shift offsets

**Table 3:** Average position errors with and without correction.

Sequence	Method	Mean Position Error (m)	(%) of Trajectory
00	VO+SeqSLAM	7.98	0.2
	VO	14.26	0.39
05	VO+SeqSLAM	5.59	0.25
	VO	9.02	0.41
06	VO+SeqSLAM	3.43	0.27
	VO	6.54	0.53

**Table 4:** Error of the ending pose with and without correction.

Sequence	Method	Position Error (m)	Heading Error (deg)
00	VO+SeqSLAM	4.5	2.5
	VO	16.71	9.96
05	VO+SeqSLAM	1.37	2.85
	VO	14.29	6.03
06	VO+SeqSLAM	3.93	1.31
	VO	18.03	3.55

**Table 5:** EKF-based fusion vs UKF-based.

Sequence	Fusion Method	Mean Position Error (m)	(%) of Trajectory
00	EKF	7.98	0.2
	UKF	7.96	0.2
05	EKF	5.59	0.25
	UKF	5.62	0.25

## Abbreviations

The following abbreviations are used in this manuscript:

GPS	Global Positioning System
SfM	Structure from Motion
VO	Visual Odometry
SeqSLAM	Sequence SLAM
SLAM	Simultaneous Localization and Mapping
KF	Kalman Filter
UKF	Unscented Kalman Filter
FP	False positive
FN	False negative
*pdf*	probability density function
